# Involvement of *CUL4A* in Regulation of Multidrug Resistance to P-gp Substrate Drugs in Breast Cancer Cells

**DOI:** 10.3390/molecules19010159

**Published:** 2013-12-24

**Authors:** Yunshan Wang, Guangxin Ma, Qin Wang, Mingxin Wen, Yangyang Xu, Xiuquan He, Pengju Zhang, Yuli Wang, Taomei Yang, Panpan Zhan, Guangwei Wei

**Affiliations:** 1 Department of Human Anatomy and Key Laboratory of Experimental Teratology, Ministry of Education, Shandong University School of Medicine, 44 Wenhua Xi Road, Jinan 250012, Shandong, China; E-Mails: wangyunshan135@126.com (Y.W.); 1987129matiancai@163.com (G.M.); wen20042302079@163.com (M.W.); xuyagnyang306@163.com (Y.X.); hxq3000@sdu.edu.cn (X.H.); sdaqyl@126.com (Y.W.); 478470886@qq.com (T.Y.); 2 International Biotechnology R&D Center, Shandong University School of Ocean, 180 Wenhua Xi Road, Weihai 264209, Shandong, China; 3 Department of Respiratory Medicine, Qilu Hospital, Shandong University, 107 Wenhua Xi Road, Jinan 250012, Shandong, China; E-Mail: wangqin8207@126.com; 4 Department of Biochemistry and Molecular Biology, Shandong University School of Medicine, 44 Wenhua Xi Road, Jinan 250012, Shandong, China; E-Mails: zhangpengjusdu@126.com (P.Z.); 531489819@qq.com (P.Z.)

**Keywords:** multidrug resistant, P-glycoprotein, *CUL4A*, breast cancer

## Abstract

*CUL4A* encodes a core component of a cullin-based E3 ubiquitin ligase complex that regulates many critical processes such as cell cycle progression, DNA replication, DNA repair and chromatin remodeling by targeting a variety of proteins for ubiquitination and degradation. In the research described in this report we aimed to clarify whether *CUL4A* participates in multiple drug resistance (MDR) in breast cancer cells. We first transfected vectors carrying *CUL4A* and specific shCUL4A into breast cancer cells and corresponding Adr cells respectively. Using reverse transcription polymerase chain reactions and western blots, we found that overexpression of *CUL4A* in MCF7 and MDA-MB-468 cells up-regulated *MDR1/*P-gp expression on both the transcription and protein levels, which conferred multidrug resistance to P-gp substrate drugs, as determined by 3-(4,5-dimethylthiazol-2-yl)-2,5-diphenyltetrazolium bromide (MTT) assays. On the other hand, silencing *CUL4A* in MCF7/Adr and MDA-MB-468/Adr cells led to the opposite effect. Moreover, ERK1/2 in *CUL4A*-overexpressing cells was highly activated and after treatment with PD98059, an ERK1/2-specific inhibitor, *CUL4A*-induced expression of *MDR1/*P-gp was decreased significantly. Lastly, immunohistochemistry in breast cancer tissues showed that P-gp expression had a positive correlation with the expression of *CUL4A* and ERK1/2. Thus, these results implied that *CUL4A* and ERK1/2 participated in multi-drug resistance in breast cancer through regulation of *MDR1/*P-gp expression.

## 1. Introduction

Breast cancer is the most frequently diagnosed cancer in woman and one of the leading causes of cancer death worldwide [1,2]. Multiple drug resistance (MDR) plays a major role in the failure of breast cancer therapy [3,4,5]. The development of MDR, in which tumor cells become resistant to a wide spectrum of anti-cancer agents with different structures or different target sites, severely limits the success of chemotherapy in breast cancer [3,4,6,7]. The mechanisms of MDR acquisition in the course of chemotherapy are numerous and complex. The human multidrug resistance 1 gene (*MDR1*) encodes the membrane-located efflux pump P-glycoprotein (P-gp). Overexpression of *MDR1*/P-gp results in an active efflux of anticancer agents from cells, thus lowering intracellular drug concentrations and inducing cancer cells to resist to chemotherapeutic drugs, especially P-gp substrate anti-cancer drugs, such as doxorubicin and paclitaxel [8,9,10,11]. Several strategies have been developed to restore chemotherapeutic sensitivity in MDR cells with limited success [7,12]. 

*CUL4A*, a newly found oncogene, is amplified in breast cancers [13]. It encodes a core component of a cullin-based E3 ubiquitin ligase complex. This complex regulates many critical processes such as cell cycle progression, DNA replication, DNA repair and chromatin remodeling by targeting a variety of proteins for ubiquitination and degradation [14,15,16,17]. In breast cancers, over-expression of *CUL4A* strongly correlates with poor prognosis [18]. Recent research showed that *CUL4A* has a relationship with MDR in prostate cancer too [19], but the relationship between *CUL4A* and MDR and related mechanisms in breast cancer are still unclear.

The mitogen-activated protein kinase (MAPK) pathway is an attractive target for therapeutic intervention in cancer due to its integral role in the regulation of cancer cell proliferation, invasiveness, and survival [20,21]. Extracellular signal-regulated kinase (ERK) is a member of the MAPK family. ERK1 and ERK2 are isoforms of the “classical” MAPK [22]. The activity of ERK1/2 has been implicated in the regulation of embryonic morphogenesis, cell proliferation, tumor transformation, and apoptosis [23,24]. It has been recently found that *CUL4A* accommodate ERK1/2 expression by adjusting H3K4 methylation level on its promoter region in PC3 cell line [19]. Moreover, it also had been reported that P-gp expression in the *MDR1*-transduced human breast cancer cell lines MCF-7/Adr and MDA-MB-231/Adr is positively regulated by the ERK1/2 pathway and blockage of this pathway can suppress cell surface P-gp expression [25]. In addition, there are several lines of evidence that modulation of ERK1/2 activation may reverse MDR in prostatic, gastric and hematopoietic cancers [26,27,28]. Therefore, we presume that *CUL4A* may regulate MDR in breast cancer through modulation of ERK1/2.

Our main purpose in the current research was to explore whether *CUL4A* participated in the regulation of MDR and whether *MDR1/*P-gp expression could be mediated by *CUL4A* via the ERK1/2 pathway. Our results suggest that overexpression of *CUL4A* in breast cancer is responsible for the required resistant to P-gp substrate drugs through regulation of *MDR1/*P-gp expression.

## 2. Results and Discussion

### 2.1. Vectors Stably Expressing CUL4A and CUL4A shRNA Caused Specific and Effective Up- or Down-Expression of CUL4A, Respectively

**Figure 1:**
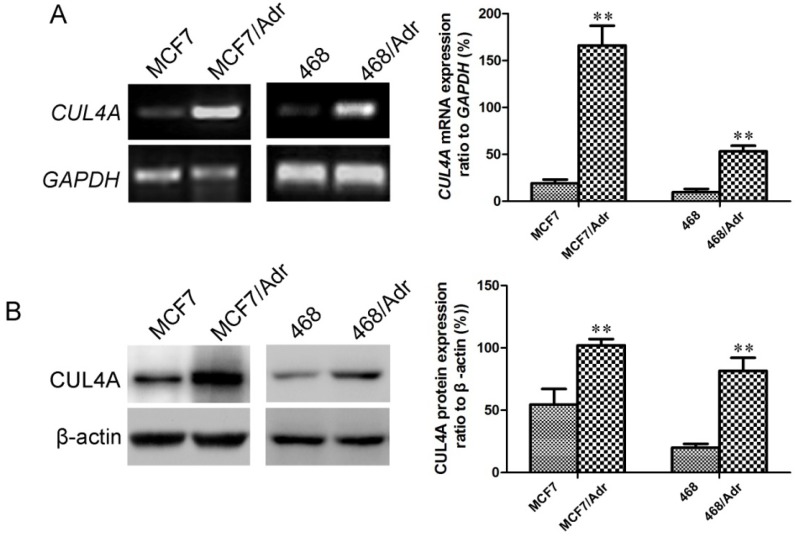
*CUL4A* mRNA and protein levels in MCF7 and MDA-MB-468 cells were up-regulated after adriamycin treatment. (**A**) *CUL4A* mRNA levels assessed by RT-PCR. (**B**) *CUL4A* protein levels assessed by Western blot. *GAPDH* mRNA and β-actin protein were served as loading controls. 468: MDA-MB-468. Bar graphs represent mean ± SEM of three independent experiments. ^**^ *p* < 0.01 *vs.* MCF7 or MDA-MB-468 cells was obtained from Student’s *t*-test.

As shown in Figure 1, after induction with adriamycin, mRNA (Figure 1A) and protein (Figure 1B) levels of *CUL4A* in MCF7/Adr and MDA-MB-468/Adr cells were much higher than those in MCF7 and MDA-MB-468 cells. 

To establish cell lines with ectopic or silencing *CUL4A* expression, MCF7 and MDA-MB-468 and their Adr cells were stably transfected with retroviruses expressing pBabe-CUL4A and pSuper-shCUL4A respectively. The transfection efficiencies of individual stable transfectant were first evaluated using RT-PCR. Relative *CUL4A* mRNA levels in each transfectant were normalized against mRNA levels of an internal control gene, *GAPDH*, carried out in different runs. 

**Figure 2:**
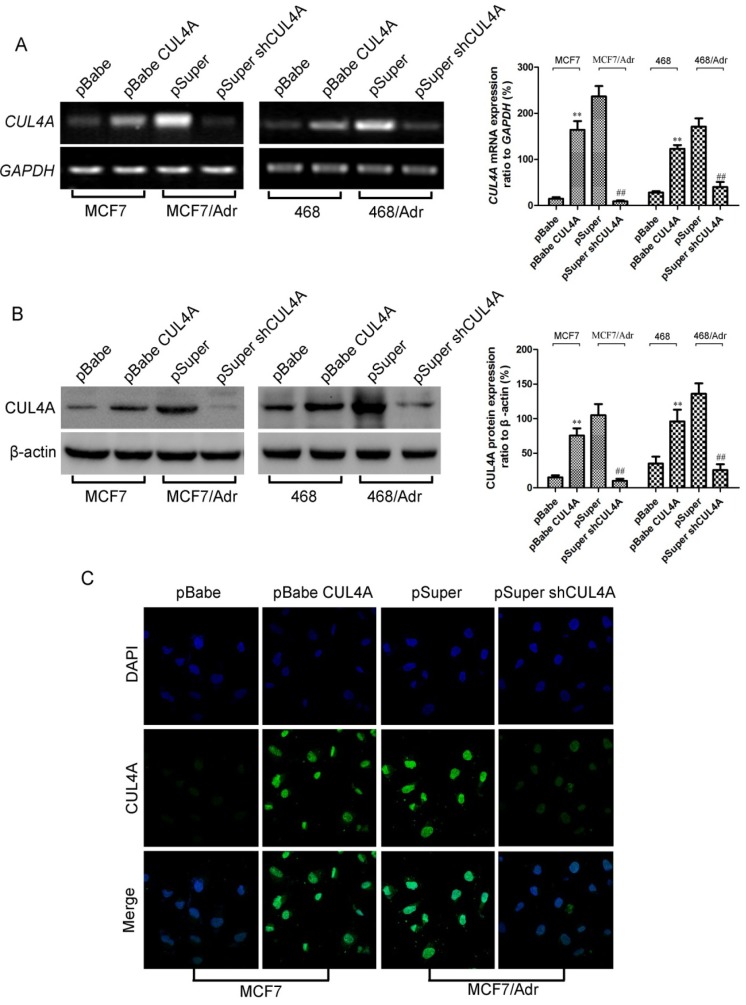
*CUL4A* mRNA and protein levels in breast cancer cells were up- or down-regulated after retroviral transfection with *CUL4A* or *CUL4A* shRNA vectors respectively. (**A**) *CUL4A* mRNA levels assessed by RT-PCR. (**B**) *CUL4A* protein levels assessed by western blot. *GAPDH* mRNA and β-actin protein were served as controls for sample loading. Bar graphs represent mean ± SEM of three independent experiments. (**C**) *CUL4A* protein assessed by immunofluorescence microscopy. Cell images were captured by Laser Scanning microscopy (magnification of ×630). 468: MDA-MB-468. ^**^ *p* < 0.01 *vs.* pBabe cells and ^##^ *P* < 0.01 *vs.* pSuper cells were obtained from student’s *t*-test.

As shown in Figure 2, we demonstrated a significant promotion or reduction of *CUL4A* transcription when compared with empty vector controls (Figure 2A). In addition, Western blot (Figure 2B) and immunofluorescence microscopy (Figure 2C) analyses showed the responsive changes in corresponding stable transfectants. The results above showed that the expression of *CUL4A* was up- or down-regulated effectively and stable cell lines with ectopic and silencing *CUL4A* expression were established by *CUL4A* and *CUL4A* specific shRNA vectors respectively.

**Figure 3:**
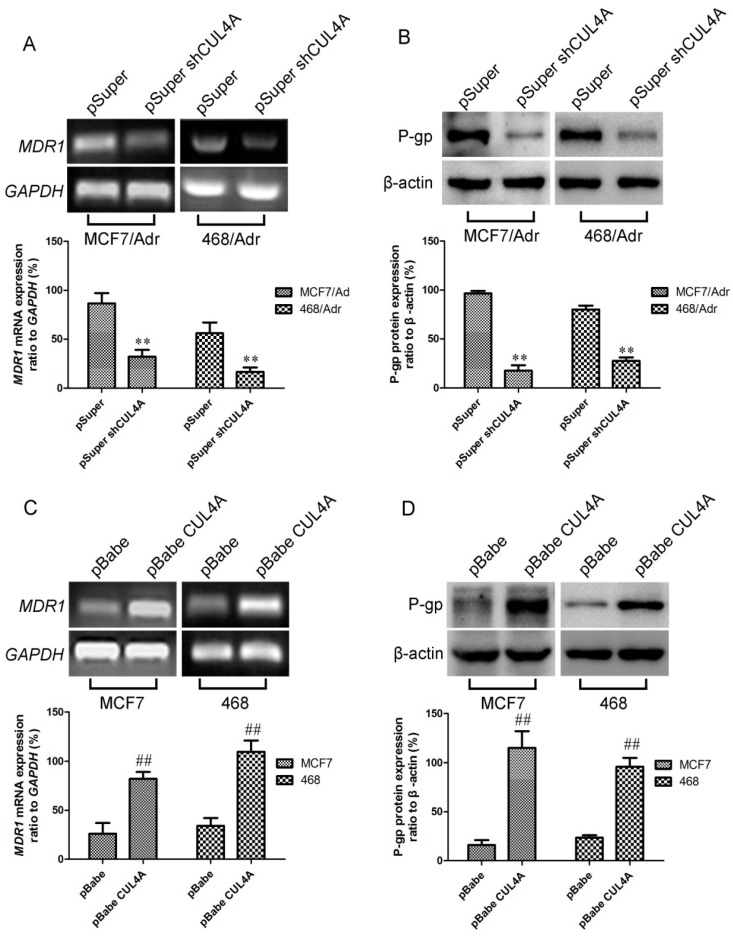
Effects of *CUL4A* on the regulation of *MDR1/*P-gp expression. Endogenous *MDR1* mRNA (**A**, **C**) and protein levels (**B**, **D**) were measured by RT-PCR and western blot, respectively. Bar graphs represent mean ± SEM of three independent experiments. 468: MDA-MB-468. ^##^ *p* < 0.01 *vs.* pBabe cells and ^**^ *p* < 0.01 *vs.* pSuper cells were obtained from Student’s *t*-test.

### 2.2. Regulation of MDR1 mRNA and Protein Levels by CUL4A in Breast Cancer Cells

To confirm the participation of *CUL4A* in regulation of *MDR1* expression, we used the above MCF7/Adr and MDA-MB-468/Adr-derived cell lines in which overexpression of *CUL4A* was reduced by stable transfection of *CUL4A*-RNAi vector (pSuper-shCUL4A). RT-PCR revealed distinct changes in *MDR1* level between *CUL4A* silencing transfectants and their empty vector controls (Figure 3A). Western blot analysis showed the corresponding reductions in protein levels (Figure 3B). To further investigate whether *CUL4A* affects *MDR1* gene expression, total RNA was obtained from ectopic *CUL4A* expressing cells, *MDR1* and *GAPDH* mRNA levels were measured by RT-PCR. As seen in Figure 3C, endogenous *MDR1* level was remarkably higher in *CUL4A* transfectants than their empty vector control transfectants (*p* < 0.05). To assess whether the *CUL4A* induced increase in *MDR1* mRNA levels were associated with a corresponding elevation in protein levels, lysates from the above cell lines were prepared and western blot was carried out. Results of multiple western blot analyses showed that P-gp levels were significantly higher in ectopic *CUL4A* expressing cells than in their empty vector control transfectants (Figure 3D).

### 2.3. Knockdown of CUL4A in MCF7/Adr Cells Resulted in Reduced Resistance to P-gp Substrate Drugs, Whereas Elevated Expression of CUL4A in MCF7 Cells Led to the Opposite Effects

*CUL4A* has been showed to be overexpressed in human MDR carcinoma cell lines, and the above results also demonstrated that *CUL4A* participated in regulation of *MDR1* gene expression. Therefore, we hypothesized that *CUL4A* would affect the sensitivity to P-gp substrate drugs. To test this hypothesis, we used *CUL4A* overexpressing and *CUL4A* silencing cells previously derived from the MCF7 and MCF7/Adr line respectively. As reported in Table 1, *CUL4A* overexpression had varying effects on drug sensitivity depending on the drug used. Interestingly, the *CUL4A* overexpression transfectant showed decreased sensitivity to P-gp substrate drugs, Taxel, vincristine (VCR) and adriamycin (ADR) (*p* < 0.05), while knockdown of *CUL4A* in MCF7/Adr cells led to the opposite effects (*p* < 0.05) when compared with their empty vector controls. The results also showed that the P-gp inhibitor LY335979 reversed *CUL4A* mediated decreased sensitivity to P-gp substrate drugs. The resistance to P-gp non-substrate drugs bleomycin (BLM) and camptothecin (Cam) remained unchanged (*p* > 0.05) after alteration of *CUL4A* expression.

**Table 1:** IC_50_ of VCR, Taxel, ADR, Cam and BLM in *CUL4A*, shCUL4A transfectants and their empty vector controls.

Drugs	MCF7 pBabe	MCF7 pBabe-CUL4A	MCF7 pBabe CUL4A+ LY335979	MCF7/Adr pSuper	MCF7/Adr pSuper-shCUL4A
VCR	0.4143 ± 0.0427	5.9716 ± 0.5329 ^*^	0.6961 ± 0.0714	7.6733 ± 1.0576	1.3982 ± 0.3155 ^#^
Taxel	0.0197 ± 0.0026	0.8659 ± 0.0691 ^*^	0.0263 ± 0.0047	0.9147 ± 0.1468	0.1685 ± 0.0138 ^#^
ADR	0.5579 ± 0.0674	17.9716 ± 1.3158 ^*^	1.2682 ± 0.4793	9.7558 ± 0.7871	2.4982 ± 0.4133 ^#^
Cam	0.0107 ± 0.0024	0.0129 ± 0.0037	0.0118 ± 0.0021	0.0112 ± 0.0019	0.0103 ± 0.0025
BLM	0.5792 ± 0.0717	0.6511 ± 0.0325	0.6392 ± 0.0592	0.5935 ± 0.0815	0.5563 ± 0.0648

ADR, adriamycin; BLM, bleomycin; Cam, camptothecin; Taxel, paclitaxel; VCR, vincristine. IC_50_ values are expressed in μM and were evaluated as reported in Materials and Methods. Standard deviations for all of the experiments carried out in triplicate were less than 5%. ^*^ *p* < 0.05 *vs.* pBabe cells; ^#^ *p* < 0.05 *vs.* pSuper cells.

### 2.4. CUL4A Activated ERK1/2 Signaling Pathway via Transcriptional Regulation and ERK1/2 is a Mediator for CUL4A Induced P-gp Expression

**Figure 4:**
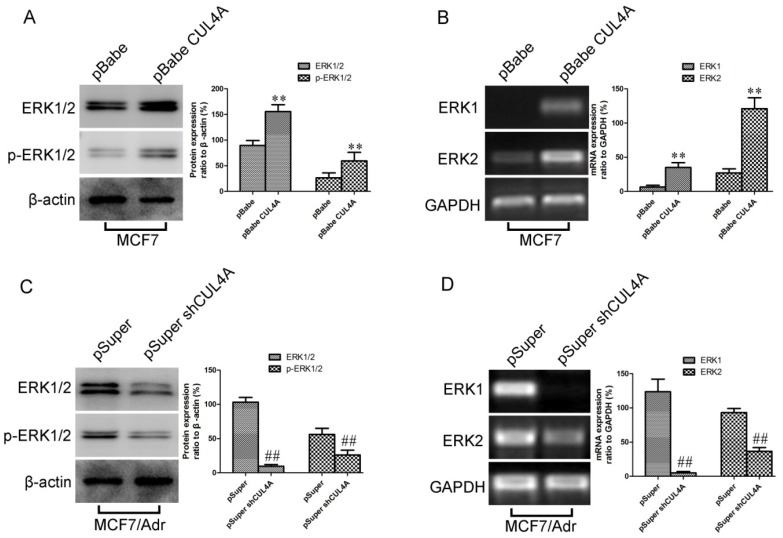
*CUL4A* activates the ERK1/2 signaling pathway. (**A**) Expression of ERK1/2 and p-ERK1/2 protein in MCF7-pBabe and MCF7-pBabe-CUL4A were assessed by western blot. (**B**) Levels of *ERK1* and *ERK2* mRNA in MCF7-pBabe and MCF7-pBabe-CUL4A were assessed by RT-PCR. (**C**) Expression of ERK1/2 and p-ERK1/2 protein in MCF7/Adr-pSuper and MCF7/Adr-pSuper-shCUL4A were assessed by western blot. (**D**) Levels of *ERK1* and *ERK2* mRNA in MCF7/Adr-pSuper and MCF7/Adr-pSuper-shCUL4A were assessed by RT-PCR. *GAPDH* mRNA and β-actin protein served as controls for sample loading. Bar graphs represent mean ± SEM of three independent experiments. ^**^ *p* < 0.01 *vs.* MCF7-pBabe cells; ^##^ *p* < 0.01 *vs.* MCF7/Adr-pSuper cells.

Previous studies have established that P-gp works in conjunction with the ERK1/2 pathway [29]. This evidence promoted us to investigate whether *CUL4A* regulates P-gp expression via the ERK signaling pathway. Phosphorylated ERK1/2 and total ERK1/2 in the MCF7 and MDA-MB-468 derived transfectants were compared to their control cells by western blot. As showed in Figure 4A and Figure S1A, the phosphorylated ERK1/2 and total levels of ERK 1/2 proteins strongly increased in cells with overexpression of *CUL4A*, while knockdown of *CUL4A* by shRNA in MCF7/Adr and MDA-MB-468/Adr cells strongly decreased the levels of both phosphorylated ERK1/2 and total ERK 1/2 proteins (Figure 4C, Figure S1C). We further found that the transcriptional levels of both *ERK1* and *ERK2* changed according to *CUL4A* level (Figure 4B,D and Figure S1B,D), indicating that *CUL4A* activates ERK1/2 signaling pathway via transcriptional regulation.

**Figure 5:**
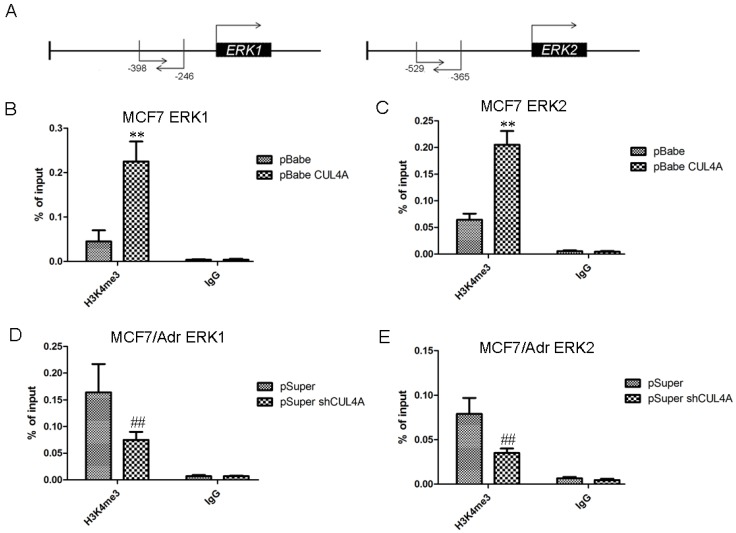
*CUL4A* regulates *ERK1/2* transcriptional expression through regulation of H3K4 methylation. (**A**) Schematic presentation of qPCR primer locations relative to the *ERK1* and *ERK2* transcriptional start sites. (**B**) ChIP-qPCR was performed to assess H3K4me3 occupancy on *ERK1* promoter in MCF7-pBabe and MCF7-pBabe-CUL4A cells. (**C**) ChIP-qPCR was performed to assess H3K4me3 occupancy on *ERK2* promoter in MCF7-pBabe and MCF7-pBabe-CUL4A cells. (**D**) ChIP-qPCR was performed to assess H3K4me3 occupancy on *ERK1* promoter in MCF7/Adr-pSuper and MCF7/Adr-pSuper-shCUL4A cells. (**E**) ChIP-qPCR was performed to assess H3K4me3 occupancy on *ERK2* promoter in MCF7/Adr-pSuper and MCF7/Adr-pSuper-shCUL4A cells. IgG was used as negative control. “Percentage of input” indicates the ratio of DNA fragment of each promoter region bound by H3K4me3 to the total amount of input DNA fragment without H3K4me3 antibody pull-down. Bar graphs represent mean ± SEM of three independent experiments. ^**^ *p* < 0.01 *vs.* MCF7-pBabe cells; ^##^ *p* < 0.01 *vs.* MCF7/Adr-pSuper cells.

Previous study showed that *CUL4A* regulates histone methylation at H3K4 [30]. Thus, we proposed that *CUL4A* may transcriptionally activate *ERK1/2* through enrichment of H3K4 trimethylation (H3K4me3) at *ERK1* and *ERK2* promoter. We showed that MCF7-CUL4A cells had higher level of H3K4me3 compared with MCF7 cells (data not show). ChIP-qPCR assays indicated that occupation of H3K4me3 on *ERK1* and *ERK2* promoters was significantly higher in MCF7-CUL4A cells compared with MCF7 cells (Figure 5B,C). Reciprocally, occupation of H3K4me3 on *ERK1* and *ERK2* promoter was significantly lower in MCF7/Adr-shCUL4A cells compared with MCF7/Adr cells (Figure 5D,E).

**Figure 6:**
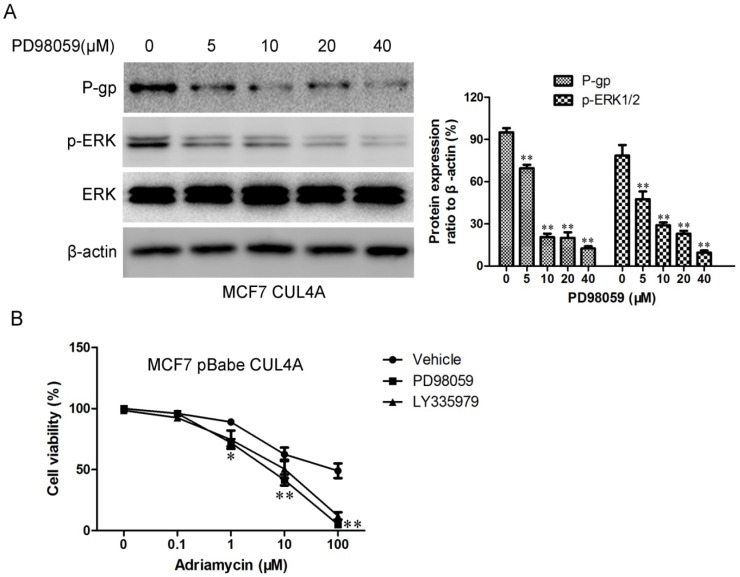
ERK1/2 inhibitor PD98059 modulated *CUL4A* regulated P-gp expression and sensitivity to P-gp substrate drug. (**A**) The levels of P-gp, ERK1/2, and p-ERK1/2 were assessed by western blot. Bar graphs represent the immunoblot signal intensity. (**B**) *CUL4A* mediated sensitivity to adriamycin after ERK1/2 inhibitor PD98059 and P-gp inhibitor LY335979 treatment in MCF7-CUL4A cells as assessed by MTT. ^*^ *p* < 0.05 and ^**^ *p* < 0.01 *vs.* vehicle controls.

We then tested the effects of ERK1/2 inhibitor on *MDR1* expression. As seen in Figure 6A, PD98059, an inhibitor of MAPK/extracellular signal regulated kinase, led to significant dose-dependent (5–40 μM) decreases in P-gp and p-ERK1/2 levels in the *CUL4A* overexpression cells. Next we monitored CUL4A-mediated sensitivity to P-gp substrate drugs after ERK1/2 inhibitor PD98059 and P-gp inhibitor LY335979 treatment in MCF7-CUL4A cells. As expected, CUL4A-mediated decreases in sensitivity to P-gp substrate drugs were attenuated by ERK1/2 inhibitor PD98059 and P-gp inhibitor LY335979 (Figure 6B). Therefore, ERK1/2 activation might be one possible mechanism for the higher P-gp expression levels in MCF7-CUL4A cells.

### 2.5. Expression and Relationship of P-gp, CUL4A and ERK1/2 in Breast Cancer Patient Samples

**Figure 7:**
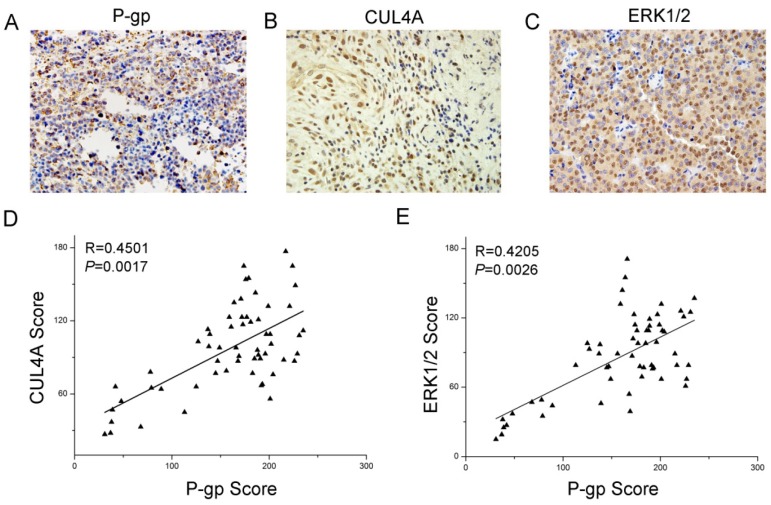
Expression and relationship of P-gp, *CUL4A* and ERK1/2 in breast cancer patient samples. Immunohistochemical analysis of P-gp (**A**), *CUL4A* (**B**) and ERK1/2 (**C**) expression in breast cancer tissues (59 cases; magnification of ×400). (**D**) P-gp expression was positively correlated with *CUL4A* expression in breast cancer tissues. (E) P-gp expression was positively correlated with ERK1/2 expression in breast cancer tissues.

To clarify the relationship among P-gp, *CUL4A* and ERK1/2 in breast cancer, immunohistochemistry was performed on 59 breast cancer tissue samples following the manufacturer’s instructions. P-gp expression had a positive ratio of 62.7% (37/59), *CUL4A* of 40.6% (24/59) and ERK1/2 of 54.2% (32/59), which had significant differences comparing with the normal breast tissues adjacent to the cancer tissues. P-gp protein was expressed in cytoplasm and cell membrane, while *CUL4A* was in the cell nucleus (Figure 7A–C). The P-gp expression showed a positive correlation with the expression of *CUL4A* (Figure 7D) and ERK1/2 (Figure 7E). These results implied that *CUL4A* and ERK1/2 participated in multi-drug resistance in breast cancer, and that it might participate through regulation of P-gp expression.

### 2.6. Discussion

Breast cancer, one of the most frequent malignancies in women worldwide, is a complex and intrinsically heterogeneous disease, which develops through the accumulation of a wide spectrum of genomic aberrations eventually leading to oncogenic properties such as immortalization, stromal invasion, and metastasis [1,2,31]. MDR has been considered a serious problem in the treatment of many cancers including breast cancer [3]. MDR is mediated by complex mechanisms and one of the major mechanisms is the expression of the *MDR1* encoded P-gp, which has been confirmed to be responsible for the resistance to several unrelated cancer chemotherapeutic drugs. Overexpression of the efflux pump MDR1/P-gp results in the decreased accumulation and toxicity of many anticancer drugs, such as doxorubicin, paclitaxel, and etoposide, leading to chemotherapeutic failure [11,32]. Although chemotherapy has improved the survival rate of breast cancer patients for decades, more than 80% patients who received chemo-drugs will eventually develop MDR after periods of treatment, leading to treatment failure. Therefore, new strategies to benefit MDR breast cancer patients are urgently required [33,34]. Although several inhibitors/modulators of MDR1/P-gp have been developed, cytotoxic effects and adverse pharmacokinetics have prohibited their use [6]. Currently available inhibitors also lack the potency to reverse the MDR phenotype completely at clinically attainable concentrations. Fortunately, molecular target therapy has shown promise in preclinical studies, based on numerous signaling pathways that play roles in the regulation of *MDR1/*P-gp expression [7,35].

*CUL4A* plays important roles in cell growth, proliferation, differentiation, longevity, metabolism, and tumor development [36,37]. In the present study we showed that there was a close correlation between *MDR1* and *CUL4A*. P-gp protein levels were elevated in *CUL4A* overexpressing human breast cancer cells. Remarkably enhanced *CUL4A* levels were observed in adriamycin resistance breast cancer cells, which have canonically overexpressed P-gp proteins. Moreover, *CUL4A* knockdown showed potent inhibitory effects on *MDR1* gene transcription and protein expression in adriamycin resistance breast cancer cells. Thus, *CUL4A* may act as a crucial factor for *MDR1* gene transcription in Adr resistant breast cancer cells.

ERK1/2 is a member of the MAPK family. The activity of ERK1/2 has been implicated in the regulation of embryonic morphogenesis, cell proliferation, malignangt transformation, and apoptosis [21]. It has been recently found that P-gp expression in the *MDR1*-transduced human breast cancer cell line MCF-7/MDR and MDA-MB-231/MDR is positively regulated by the ERK pathway and blockade of the MEK-ERK pathway can suppress cell surface P-gp expression by promoting its degradation [25]. ERK1 or ERK2 might be a potential drug target for circumventing MDR hepatocellular carcinoma cells [38]. In addition, there are several lines of evidence that modulation of ERK1/2 activation may reverse MDR in prostatic, gastric and hematopoietic cancers [26,27,28].

It has been reported that *CUL4A* is essential for ubiquitination of several well-defined tumor suppressors, including p21, p27, DDB2, and p53 [39,40,41,42]. The present study suggests that *CUL4A* activates ERK1/2 pathway. Overexpression of *CUL4A* dramatically increased ERK1/2 protein levels while knockdown of *CUL4A* remarkably decreased ERK1/2 activity. Surprisingly our results also suggested that the regulation of ERK1/2 by *CUL4A* is through transcription regulation rather than ubiquitination. Some studies have showed that CUL4A regulates histone methylation. We further showed that *CUL4A* upregulates ERK1/2 expression through enrichment of trimethylated H3K4 on *ERK1* and *ERK2* promoters. Importantly, *CUL4A*-induced malignant phenotype can be partially reversed by ERK1/2 inhibitor PD98059, which further suggests that *CUL4A* exerts the oncogenic effect in breast cancer through upregulation of ERK1/2. We also found that the ERK1/2 inhibitor PD98059 led to significant dose-dependent inhibition of P-gp expression in the *CUL4A* overexpression cells. Next we monitored CUL4A-mediated sensitivity to P-gp substrate drugs after ERK1/2 inhibitor PD98059 treatment in MCF7-CUL4A cells. As expected, *CUL4A*-mediated decreased sensitivity to P-gp substrate drugs were attenuated by ERK1/2 inhibitor PD98059. Therefore, ERK1/2 activation might be one possible mechanism for the *CUL4A* induced P-gp expression levels in MCF7-CUL4A cells.

To our knowledge, this is the first study to show that *CUL4A* plays a functional role in breast cancer multidrug resistant. *CUL4A* was overexpressed in the multidrug resistant breast cancer cell, and silencing *CUL4A* caused the reverse of multidrug resistance. *CUL4A* appeared to mediate multidrug resistance by regulating the ERK1/2 pathway, thereby leading to the observed effect on *MDR1* transcription in breast cancer cells. With the limitations of the *in vitro* model system, our findings only indicated the involvement of *CUL4A* in the ERK1/2 pathway regulating multidrug resistance in breast cancer. However, whether relapsed breast cancer patients are subject to an increase of *CUL4A* and *MDR1/*P-gp expression after chemotherapy remains unknown. Further investigation *in vivo* and of patient specimens is required to confirm the regulation, and to verify whether overexpression of *CUL4A* in breast cancer cells may be used as a potential biomarker for predicting sensitivity to chemotherapy. Our research shed light on identifying *CUL4A* as a new molecular target for reversing multidrug resistance in breast cancer.

## 3. Experimental Section

### 3.1. Cell Culture and Establishment of MCF7/Adr and MDA-MB-468/Adr Cells

Human mammary carcinoma cell line MCF7 and MDA-MB-468 were obtained from the American Type Culture Collection (ATCC, Manassas, VA, USA). Their MDR counterparts, MCF7/Adr and MDA-MB-468/Adr cells were generated by sequential exposure to increasing concentrations of Adriamycin (Sigma, St. Louis, MO, USA). Cells were cultured in RPMI 1640 (Gibco, Grand Island, NY, USA) containing 10% fetal bovine serum (FBS), 100 units/mL penicillin, and 100 μg/mL streptomycin at 37 °C in a humidified atmosphere containing 5% CO2. For consistent *MDR-1* gene expression, MCF7/Adr and MDA-MB-468/Adr cells were maintained in the presence of adriamycin (Sigma, USA).

### 3.2. Establishment of CUL4A Stable Expression and CUL4A Knockdown Cell Lines

pBabe.puro retroviral construct containing human *CUL4A* cDNA and pSuper.retro.puro with shRNA against human *CUL4A* were prepared as described previously [36]. The generation of retrovirus supernatants and transfection of breast cancer cells were conducted as described previously [36]. Infected cells were selected by adding 2 μg/mL puromycin to the culture medium for 48 hrs and then maintained in complete medium with 0.5 μg/mL puromycin. Empty retroviral vector infected stable cell lines were also produced by the above protocols. The expression of *CUL4A* was confirmed by RT-PCR and Western blot analyses.

### 3.3. Reverse Transcription and Quantitative Real-Time Polymerase Chain Reaction

Total RNA of transfected cells and their control cells was extracted using Tripure isolation reagent (Sangon, Shanghai, China) according to the manufacturer’s instructions. The quality and yield of the RNA samples were determined by ultraviolet spectrophotometer. Total RNAs (1 μg) were reverse transcribed to cDNA (20 μL) using PrimeScriptTM RT Kit (TaKaRa, Dalian, China) according to the manufacturer’s instructions. PCR reaction was conducted with 2 μL cDNA sample, 0.4 μL forward primer (10 μmol/L), 0.4 μL reverse primer (10 μmol/L), 11.2 μL RNase-free water, and 6 μL 2× EsayTaq PCR SuperMix (TransGen Biotech, Beijing, China). The primers were used as follows: *CUL4A* (forward, 5'-cagcggctctgattacagacctcg-3'; reverse, 5'-gtcttcacaggcctgacgcagt-3'); *MDR1* (forward, 5'-cccatcattgcaatagcagg-3'; reverse, 5'-gttcaaacttctgctcctga-3'); *ERK1* (forward, 5'-agtcagactccaaagcccttgacc-3'; reverse, 5'-aaggtgccatggaacaggctgt-3'); *ERK2* (forward, 5'-aaggtgccatggaacaggctgt-3'; reverse, 5'-tcctctgagcccttgtcctgac-3'); *GAPDH* (forward, 5'-acccagaagactgtggatgg-3'; reverse, 5'-acgcctgcttcaccaccttc-3'). PCR reaction was performed using the following cycle parameters: 95 °C for 5 min, (94 °C for 30 s, 56 °C for 30 s, 72 °C for 45 s) for 30 cycles, 72 °C for 7 min. RT-PCR products were separated on 2% agarose gels. After stained with ethidium bromide, gel images were photographed with a ChemiImager TM 4400. RT-PCR was performed at least three times for each sample. 

### 3.4. Western Blot

Cells were harvested and lysed in lysis buffer (0.5% Nonidet P-40, 10 mM Tris, pH 7.4, 150 mM NaCl, 1 mM EDTA, 1 mM Na_3_VO_4_) containing protease inhibitors (1 mM phenylmethylsulfonyl fluoride [PMSF]). Cell lysates (50 μg/well) were then subjected to standard sodium dodecyl sulphate polyacrylamide gel electrophoresis (SDS-PAGE). For Western blotting analysis, proteins were transferred to polyvinylidene fluoride (PVDF) membranes, and then blocked in 5% non-fat milk in 10 mM Tris, pH 7.5, 100 mMNaCl, 0.1% (w/v) Tween 20 for 1 h. The membranes were first incubated with antibodies against β-actin (Sigma), *CUL4A* (Santa Cruz, Paso Robles, CA, USA), P-gp (Chemicon, Billerica, MA, USA), phosphor specific and total *ERK1/2* (Cell Signaling), respectively, overnight at 4 °C, followed by 1 h incubation with the appropriate secondary antibody. For quantification of protein expression levels, AlexaFluor-700/800 nm secondary conjugates were used and PVDF membranes were analyzed using the Odyssey Infra-Red Imaging System and software (Li-Cor BioSciences, Lincoln, NE, USA) according to the manufacturer’s instructions.

### 3.5. Confocal Immunofluorescence Microscopy

Cells were plated on culture slides (Costar, Manassas, VA, USA). After 24 hrs, the cells were rinsed with phosphate buffered saline (PBS) and fixed with 4% paraformaldehyde in PBS, and cell membrane was permeabilized using 0.5% Triton X-100. These cells were then blocked for 30 min in 5% BSA in PBS and then incubated with primary antibody of *CUL4A* in 5% BSA overnight at 4 °C. After three washes in PBS, the slides were incubated for 1 h in the dark with FITC-conjugated secondary goat anti-mouse (Invitrogen, Grand Island, NY, USA). After three further washes, the slides were stained with DAPI for 5 min to visualize the nuclei, and examined using a Carl Zeiss confocal imaging system (LSM 780) (Carl Zeiss, Jena, Germany).

### 3.6. Drug Sensitivity Assay

To assess their multidrug chemosensitivity, transfected cells and their corresponding control cells were plated in 96-well plates at a density of 5 × 10^3^ cells/well and further incubated for 24 h. The medium was then removed and replaced with fresh medium containing paclitaxel (Sigma), vincristine (Sigma), adriamycin (Sigma), bleomycin (Sigma), and camptothecin (Sigma) respectively, with varying concentrations for another 48 h. After that, cells were stained with 20 μL sterile MTT dye (3-[4,5-dimethylthiazol-2-yl]-2,5-diphenyltetrazolium bromide, 5 mg/mL; Sigma) at 37 °C for 4 h followed by removing the culture medium and mixing with 150 μL of dimethylsulfoxide (DMSO) thoroughly for 10 min. Spectrometric absorbance at 490 nm was measured with a microplate reader. Each group contained three wells and was repeated three times. The IC_50_ value was determined by the dose of drug that caused 50% cell viability.

### 3.7. Chromatin Immunoprecipitation (ChIP)-qPCR

Chromatin Immunoprecipitation kit (Cat. 17-371) was purchased from Millipore Corporation (Billerica, MA, USA) and ChIP experiments were carried out essentially as described [43] with modifications. Cells were crosslinked with 1% formaldehyde for 10 min at room temperature. Crosslinking was stopped by the addition of glycine to a final concentration of 0.125 M. Fixed cells were lysed in lysis buffer (1% SDS, 10 mM EDTA, 50 mM Tris-HCl, pH 8.0) and sonicated. An aliquot of 60 mg was reserved as input. For immunoprecipitation, 600 mg of protein were diluted in dilution buffer (1%Triton X-100, 2 mM EDTA, 150 mM NaCl and 20 mM Tris-HCl, pH 8.0, containing protease inhibitors), and precleared with 60 mL of A/G plus-agarose. The antibodies used for the immunoprecipitation was against Histone H3 (tri methyl 4k). Immune complexes were precipitated with A/G plus-agarose and washed sequentially with low-salt immune complex wash buffer (0.1% SDS, 1% Triton X-100, 2 mM EDTA, 20 mM Tris-HCl, pH 8.1, 150 mM NaCl), high-salt immune complex wash buffer (0.1% SDS 1% Triton X-100, 2 mM EDTA, 20 mM Tris-HCl, pH8.1, 500 mM NaCl), LiCl immune complex wash buffer (0.25 M LiCl, 1% NP-40, 1% deoxycholate-Na, 1 mM EDTA, 10 mM Tris-HCl, pH8.1), and TE buffer, and then eluted in elution buffer (1% SDS, 0.1 M NaHCO3). All samples, including inputs, were decrosslinked, treated with proteinase K, and DNA was extracted with phenol–chloroform and resuspended in TE buffer. Real-time PCR and data collection were performed with an ABI PRISM 7900HT sequence detection system. The primers used for detection of promoters after ChIP as follows: *ERK1* (forward, 5'-gccgaacctcccggtgacct-3'; reverse, 5'-gggcctggagctgtcacgtg-3'); *ERK2* (forward, 5'-gtcaccagcaccaccaacctgt-3'; reverse, 5'-gtgactccttctcctggtgacca-3'); *GAPDH* (forward, 5'-agtcagactccaaagcccttgacc-3'; reverse, 5'-aaggtgccatggaacaggctgt-3').

### 3.8. Immunohistochemistry

Breast cancer tissues were obtained from 59 patients at Qilu Hospital (Jinan, China). All experiments were approved by the Ethics Committee of Qilu Hospital and informed consent was obtained from all patients prior to specimen collection. Surgically excised tumor specimens were fixed with 10% neutral formalin, embedded in paraffin, and 4-μm thick sections were prepared. Immunostaining was performed using the avidin-biotin-peroxidase complex method (Ultrasensitive^TM^, Fuzhou, China). The sections were deparaffinized in xylene, rehydrated with graded alcohol, and then boiled in 0.01 M citrate buffer (pH 6.0) for 2 min with an autoclave. Hydrogen peroxide (0.3%) was applied to block endogenous peroxide activity, and the sections were incubated with normal goat serum to reduce nonspecific binding. Tissue sections were incubated with *CUL4A* rabbit polyclonal antibody (1:100 dilution), P-gp mouse monoclonal antibody (1:150 dilution), *ERK1/2* monoclonal antibody (1:100 dilution). Mouse immunoglobulin (at the same concentration of the antigen specific antibody) was used as a negative control. Staining for both antibodies was performed at room temperature for 2 h. Biotinylated goat antimouse serum IgG was used as a secondary antibody. After washing, the sections were incubated with streptavidin-biotin conjugated with horseradish peroxidase, and the peroxidase reaction was developed with 3, 30-diaminobenzidine tetrahydrochloride. Two independent, blinded investigators examined all tumor slides randomly. Five views were examined per slide, and 100 cells were observed per view at 400× magnification. Scores for *CUL4A*, P-gp and *ERK1/2* staining were calculated based on staining intensity (0, below the level of detection; 1, weak; 2, moderate; and 3, strong) and the percentage of cells staining at each intensity level (0%–100%). The final score was calculated by multiplying the intensity score by the percentage, producing a scoring range of 0 to 300.

### 3.9. Statistical Analysis

Statistical analysis was performed using SPSS 16.0 and GraphPad Prism 5 (GraphPad Software, Inc., San Diego, CA, USA). All data were presented as mean ± SEM, and the Student’s *t*-test was used to determine statistical significance. Comparisons between two groups were performed using the paired *t*-test. *p* < 0.05 was considered statistically significant.

## 4. Conclusions

In summary, our work confirms that *CUL4A* regulates the expression of *MDR1* via an *ERK1/2* dependent signaling pathway in breast cancer cells, which is consistent with their alterations in cellular multidrug resistance *in vitro*. 

## Supplementary Materials

Supplementary materials can be accessed at: http://www.mdpi.com/1420-3049/19/1/159/s1.

## References

[1] Forouzanfar M.H., Foreman K.J., Delossantos A.M., Lozano R., Lopez A.D., Murray C.J., Naghavi M. (2011). Breast and cervical cancer in 187 countries between 1980 and 2010: A systematic analysis. Lancet.

[2] Siegel R., Naishadham D., Jemal A. (2012). Cancer statistics, 2012. CA Cancer J. Clin..

[3] Kuo M.T. (2007). Roles of multidrug resistance genes in breast cancer chemoresistance. Adv. Exp. Med. Biol..

[4] Taheri M., Mahjoubi F., Omranipour R. (2010). Effect of MDR1 polymorphism on multidrug resistance expression in breast cancer patients. Genet. Mol. Res..

[5] Wind N.S., Holen I. (2011). Multidrug resistance in breast cancer: From *in vitro* models to clinical studies. Int. J. Breast Cancer..

[6] Patwardhan G., Gupta V., Huang J., Gu X., Liu Y.Y. (2010). Direct assessment of P-glycoprotein efflux to determine tumor response to chemotherapy. Biochem. Pharmacol..

[7] Donmez Y., Gunduz U. (2011). Reversal of multidrug resistance by small interfering RNA (siRNA) in doxorubicin-resistant MCF-7 breast cancer cells. Biomed. Pharmacother..

[8] Haber M., Bordow S.B., Haber P.S., Marshall G.M., Stewart B.W., Norris M.D. (1997). The prognostic value of MDR1 gene expression in primary untreated neuroblastoma. Eur. J. Cancer.

[9] Norris M.D., Bordow S.B., Haber P.S., Marshall G.M., Kavallaris M., Madafiglio J., Cohn S.L., Salwen H., Schmidt M.L., Hipfner D.R. (1997). Evidence that the MYCN oncogene regulates MRP gene expression in neuroblastoma. Eur. J. Cancer.

[10] Keppler D. (2011). Multidrug resistance proteins (MRPs, ABCCs): Importance for pathophysiology and drug therapy. Handb. Exp. Pharmacol..

[11] Kimura Y., Morita S.Y., Matsuo M., Ueda K. (2007). Mechanism of multidrug recognition by MDR1/ABCB1. Cancer Sci..

[12] Ludwig J.A., Szakacs G., Martin S.E., Chu B.F., Cardarelli C., Sauna Z.E., Caplen N.J., Fales H.M., Ambudkar S.V., Weinstein J.N. (2006). Selective toxicity of NSC73306 in MDR1-positive cells as a new strategy to circumvent multidrug resistance in cancer. Cancer Res..

[13] Chen L.C., Manjeshwar S., Lu Y., Moore D., Ljung B.M., Kuo W.L., Dairkee S.H., Wernick M., Collins C., Smith H.S. (1998). The human homologue for the Caenorhabditis elegans cul-4 gene is amplified and overexpressed in primary breast cancers. Cancer Res..

[14] Shiyanov P., Nag A., Raychaudhuri P. (1999). Cullin 4A associates with the UV-damaged DNA-binding protein DDB. J. Biol. Chem..

[15] Gupta A., Yang L.X., Chen L. (2002). Study of the G2/M cell cycle checkpoint in irradiated mammary epithelial cells overexpressing Cul-4A gene. Int. J. Radiat. Oncol. Biol. Phys..

[16] Li B., Yang F.C., Clapp D.W., Chun K.T. (2003). Enforced expression of CUL-4A interferes with granulocytic differentiation and exit from the cell cycle. Blood.

[17] Sugasawa K. (2006). DNA repair pathways involving Cul4A ubiquitin ligases. Tanpakushitsu Kakusan Koso..

[18] Schindl M., Gnant M., Schoppmann S.F., Horvat R., Birner P. (2007). Overexpression of the human homologue for Caenorhabditis elegans cul-4 gene is associated with poor outcome in node-negative breast cancer. Anticancer Res..

[19] Ren S., Xu C., Cui Z., Yu Y., Xu W., Wang F., Lu J., Wei M., Lu X., Gao X. (2012). Oncogenic CUL4A determines the response to thalidomide treatment in prostate cancer. J. Mol. Med. (Berl).

[20] Panka D.J., Atkins M.B., Mier J.W. (2006). Targeting the mitogen-activated protein kinase pathway in the treatment of malignant melanoma. Clin. Cancer Res..

[21] Johnson G.L., Lapadat R. (2002). Mitogen-activated protein kinase pathways mediated by ERK, JNK, and p38 protein kinases. Science.

[22] Yang S.H., Sharrocks A.D., Whitmarsh A.J. (2013). MAP kinase signalling cascades and transcriptional regulation. Gene.

[23] Kohno M., Tanimura S., Ozaki K. (2011). Targeting the extracellular signal-regulated kinase pathway in cancer therapy. Biol. Pharm. Bull..

[24] Wei F., Yan J., Tang D. (2011). Extracellular signal-regulated kinases modulate DNA damage response—A contributing factor to using MEK inhibitors in cancer therapy. Curr. Med. Chem..

[25] Katayama K., Yoshioka S., Tsukahara S., Mitsuhashi J., Sugimoto Y. (2007). Inhibition of the mitogen-activated protein kinase pathway results in the down-regulation of P-glycoprotein. Mol. Cancer Ther..

[26] Kisucka J., Barancik M., Bohacova V., Breier A. (2001). Reversal effect of specific inhibitors of extracellular-signal regulated protein kinase pathway on P-glycoprotein mediated vincristine resistance of L1210 cells. Gen. Physiol. Biophys..

[27] Lin J.C., Chang S.Y., Hsieh D.S., Lee C.F., Yu D.S. (2005). Modulation of mitogen-activated protein kinase cascades by differentiation-1 protein: Acquired drug resistance of hormone independent prostate cancer cells. J. Urol..

[28] Li Y., Li S., Han Y., Liu J., Zhang J., Li F., Wang Y., Liu X., Yao L. (2008). Calebin-A induces apoptosis and modulates MAPK family activity in drug resistant human gastric cancer cells. Eur. J. Pharmacol..

[29] McCubrey J.A., Steelman L.S., Chappell W.H., Abrams S.L., Wong E.W., Chang F., Lehmann B., Terrian D.M., Milella M., Tafuri A. (2007). Roles of the Raf/MEK/ERK pathway in cell growth, malignant transformation and drug resistance. Biochim. Biophys. Acta.

[30] Higa L.A., Wu M., Ye T., Kobayashi R., Sun H., Zhang H. (2006). CUL4-DDB1 ubiquitin ligase interacts with multiple WD40-repeat proteins and regulates histone methylation. Nat. Cell. Biol..

[31] Di Cosimo S., Baselga J. (2010). Management of breast cancer with targeted agents: Importance of heterogeneity [corrected]. Nat. Rev. Clin. Oncol..

[32] Li Q.Q., Wang W.J., Xu J.D., Cao X.X., Chen Q., Yang J.M., Xu Z.D. (2007). Involvement of CD147 in regulation of multidrug resistance to P-gp substrate drugs and *in vitro* invasion in breast cancer cells. Cancer Sci..

[33] Xu J.W., Li Q.Q., Tao L.L., Cheng Y.Y., Yu J., Chen Q., Liu X.P., Xu Z.D. (2011). Involvement of EGFR in the promotion of malignant properties in multidrug resistant breast cancer cells. Int. J. Oncol..

[34] Amiri-Kordestani L., Basseville A., Kurdziel K., Fojo A.T., Bates S.E. (2012). Targeting MDR in breast and lung cancer: Discriminating its potential importance from the failure of drug resistance reversal studies. Drug Resist. Updat..

[35] Borowski E., Bontemps-Gracz M.M., Piwkowska A. (2005). Strategies for overcoming ABC-transporters-mediated multidrug resistance (MDR) of tumor cells. Acta Biochim. Pol..

[36] Hung M.S., Mao J.H., Xu Z., Yang C.T., Yu J.S., Harvard C., Lin Y.C., Bravo D.T., Jablons D.M., You L. (2011). Cul4A is an oncogene in malignant pleural mesothelioma. J. Cell. Mol. Med..

[37] Lee J., Zhou P. (2012). Pathogenic role of the CRL4 ubiquitin ligase in human disease. Front. Oncol..

[38] Yan F., Wang X.M., Pan C., Ma Q.M. (2009). Down-regulation of extracellular signal-regulated kinase 1/2 activity in P-glycoprotein-mediated multidrug resistant hepatocellular carcinoma cells. World J. Gastroenterol..

[39] Nag A., Bagchi S., Raychaudhuri P. (2004). Cul4A physically associates with MDM2 and participates in the proteolysis of p53. Cancer Res..

[40] Bondar T., Kalinina A., Khair L., Kopanja D., Nag A., Bagchi S., Raychaudhuri P. (2006). Cul4A and DDB1 associate with Skp2 to target p27Kip1 for proteolysis involving the COP9 signalosome. Mol. Cell. Biol..

[41] Nishitani H., Shiomi Y., Iida H., Michishita M., Takami T., Tsurimoto T. (2008). CDK inhibitor p21 is degraded by a proliferating cell nuclear antigen-coupled Cul4-DDB1Cdt2 pathway during S phase and after UV irradiation. J. Biol. Chem..

[42] El-Mahdy M.A., Zhu Q., Wang Q.E., Wani G., Praetorius-Ibba M., Wani A.A. (2006). Cullin 4A-mediated proteolysis of DDB2 protein at DNA damage sites regulates *in vivo* lesion recognition by XPC. J. Biol. Chem..

[43] Ang Y.S., Tsai S.Y., Lee D.F., Monk J., Su J., Ratnakumar K., Ding J., Ge Y., Darr H., Chang B. (2011). Wdr5 mediates self-renewal and reprogramming via the embryonic stem cell core transcriptional network. Cell.

