# Supplementary material for: Involvement of CUL4A in Regulation of Multidrug Resistance to P-gp Substrate Drugs in Breast Cancer Cells
Source: Molecules. 2013 Dec 24;19(1):159–76. doi: 10.3390/molecules19010159 (PMC6271407; doi:10.3390/molecules19010159)

Supplementary Materials

Figure S1. CUL4A activates *ERK1/2* signaling pathway. (**A**) The expression of *ERK1/2* and p-*ERK1/2* protein in MDA-MB-468-pBabe and MDA-MB-468-pBabe-CUL4A were assessed by Western blot. (**B**) The expression of ERK1 and ERK2 mRNA in MDA-MB-468-pBabe and MDA-MB-468-pBabe-CUL4A were assessed by RT-PCR. (**C**) The expression of *ERK1/2* and p-*ERK1/2* protein in MDA-MB-468/Adr-pSuper and MDA-MB-468/Adr-pSuper -shCUL4A were assessed by Western blot. (**D**) The expression of ERK1 and ERK2 mRNA in MDA-MB-468/Adr-pSuper and MDA-MB-468/Adr-pSuper -shCUL4A were assessed by RT-PCR. The expression of GAPDH mRNA and β-actin protein were also examined and served as controls for sample loading. Bar graphs represent mean ± SEM of three independent experiments. 468: MDA-MB-468. $^{\#\#}p < 0.01$ *vs.* pBabe cells; $^{**}p < 0.01$ *vs.* pSuper cells.

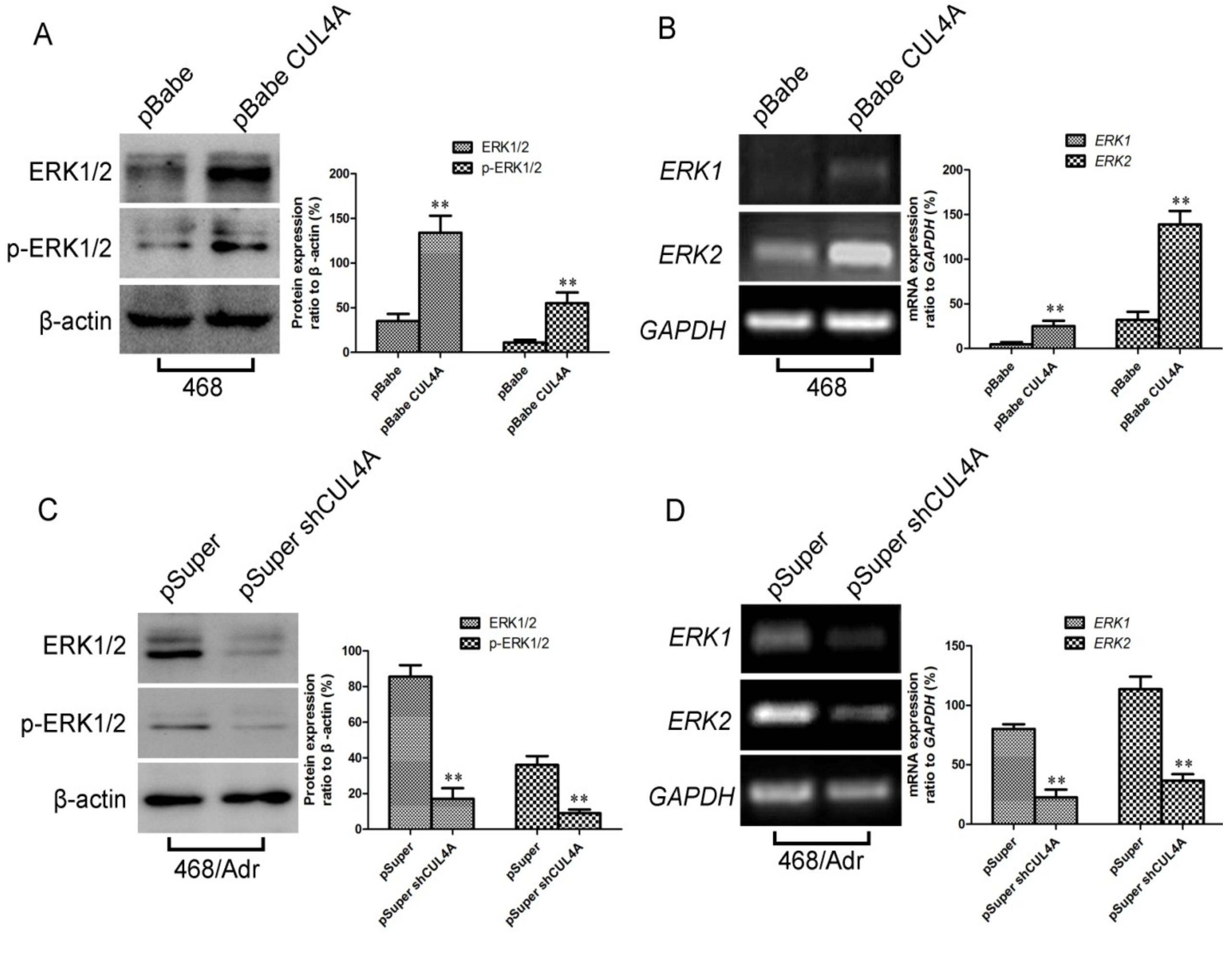

Supplement: Supplementary file 1 [file molecules-19-00159-s001.pdf]
